# Collagen Induced Arthritis in DBA/1J Mice Associates with Oxylipin Changes in Plasma

**DOI:** 10.1155/2015/543541

**Published:** 2015-10-29

**Authors:** Min He, Eduard van Wijk, Ruud Berger, Mei Wang, Katrin Strassburg, Johannes C. Schoeman, Rob J. Vreeken, Herman van Wietmarschen, Amy C. Harms, Masaki Kobayashi, Thomas Hankemeier, Jan van der Greef

**Affiliations:** ^1^Analytical BioSciences, LACDR, Leiden University, P.O. Box 9502, 2300 RA Leiden, Netherlands; ^2^Sino-Dutch Centre for Preventive and Personalized Medicine, Leiden University, P.O. Box 9502, 2300 RA Leiden, Netherlands; ^3^SU Biomedicine, Utrechtseweg 48, 3700 AJ Zeist, Netherlands; ^4^Discovery Sciences, Janssen Research & Development, Turnhoutseweg 30, 2340 Beerse, Belgium; ^5^TNO Innovation for Life, P.O. Box 360, 3700 AJ Zeist, Netherlands; ^6^Tohoku Institute of Technology, Sendai 982-8577, Japan

## Abstract

Oxylipins play important roles in various biological processes and are considered as mediators of inflammation for a wide range of diseases such as rheumatoid arthritis (RA). The purpose of this research was to study differences in oxylipin levels between a widely used collagen induced arthritis (CIA) mice model and healthy control (Ctrl) mice. DBA/1J male mice (age: 6-7 weeks) were selected and randomly divided into two groups, namely, a CIA and a Ctrl group. The CIA mice were injected intraperitoneally (i.p.) with the joint cartilage component collagen type II (CII) and an adjuvant injection of lipopolysaccharide (LPS). Oxylipin metabolites were extracted from plasma for each individual sample using solid phase extraction (SPE) and were detected with high performance liquid chromatography/tandem mass spectrometry (HPLC-ESI-MS/MS), using dynamic multiple reaction monitoring (dMRM). Both univariate and multivariate statistical analyses were applied. The results in univariate Student's *t*-test revealed 10 significantly up- or downregulated oxylipins in CIA mice, which were supplemented by another 6 additional oxylipins, contributing to group clustering upon multivariate analysis. The dysregulation of these oxylipins revealed the presence of ROS-generated oxylipins and an increase of inflammation in CIA mice. The results also suggested that the collagen induced arthritis might associate with dysregulation of apoptosis, possibly inhibited by activated NF-*κ*B because of insufficient PPAR-*γ* ligands.

## 1. Introduction

Rheumatoid arthritis (RA) is a chronic, destructive autoimmune disease which involves primarily the joints in the extremities. The disease is characterized by the destruction of the cartilage in the joints and inflammation of the synovium. This local immune response is characterized by both cell-mediated and humoral immune factors. CD4+ T cells and activated B cells are present in the synovium together with cytokines such as interleukins (e.g., IL-1 and IL-6), tumor necrosis factor (TNF*α*), and interferon gamma (IF-*γ*) [1–3]. Recent studies have shown an important role of fibroblasts-like synovial cells in the pathophysiology of RA [4–6]. Upon proinflammatory stimuli and in combination with genetic and epigenetic/environmental factors, these cells, normally responsible for proper composition of the synovial fluid and extracellular matrix, transform into an aggressive phenotype. This phenotype is characterized by a reduced ability to undergo apoptosis [7–12], the production of extracellular enzymes like collagenase and metalloproteases responsible for the destruction of the joints [13, 14], and the secretion of (pro-/anti-) inflammatory cytokines, chemokines, proangiogenic factors, and oxylipins [15–17]. Due to local hypoxia, the formation of reactive oxygen and nitrogen species is promoted [18–21].

Although the role of cytokine/chemokine triggered signal transduction pathways such as MAP kinase and nuclear factor-kappa B (NF-*κ*B) in the pathophysiology of RA has been subject of extensive research, the role of oxylipins is less well understood. Oxylipins are bioactive lipid mediators synthesized from omega-6 polyunsaturated fatty acid such as arachidonic acid (AA), linoleic acid (LA), and dihomo-gamma-linolenic acid (DGLA) and omega-3 polyunsaturated fatty acid like eicosapentenoic acid (EPA), docosahexanoic acid (DHA), and alpha-linolenic acid (ALA) upon liberation from membrane bound phospholipids by activation of phospholipase A2 and subsequent oxidation by cyclooxygenase (COX), lipoxygenase (LOX), and cytochrome P450 epoxygenase (CYP450) systems [22]. This leads to the formation of, over at least hundred, bioactive oxylipins such as prostaglandins (PG), leukotrienes (LT), thromboxanes (TBX), hydroxyeicosatetraenoic acids (HETEs), and epoxyeicosatrienoic acids (EpETrEs). They can act on both local and distant targets by secretion into the circulation system of body. AA is the substrate of proinflammatory lipid mediators while EPA and DHA derived lipid mediators are anti-inflammatory such as resolvins and protectins playing a role in the resolution of inflammation [23]. Nonenzymatic oxidation of polyunsaturated fatty acids produces the closely related bioactive lipids mediators like, for example, isoprostanes, HETEs, and HDoHEs, indicators of oxidative stress [24–29]. Therefore, investigation of the changes of oxylipins in RA animal models will certainly contribute to the understanding of biochemical events in RA research.

Metabolomics is an important and rapidly emerging field of technology enabling the comprehensive analysis of a large number of metabolites associated with disease phenotypes. We have applied a metabolomics approach using a LC-MS based platform combined with elaborate statistical methods to analyze oxylipins in a validated model of RA that is collagen induced arthritis in mice. Our results point to a diminished anti-inflammatory response and increased oxidative stress in the RA induced situation.

## 2. Materials and Method

### 2.1. Chemicals

Methanol (MeOH), acetonitrile (ACN), isopropanol (IPA), ethyl-acetate (EtOAC), and purified water were purchased from Biosolve (Netherlands). All reagents used during the HPLC-MS/MS experiments were ultra-performance liquid chromatography grade (UPLC). Acetic acid was purchased from Sigma-Aldrich (St. Louis, Mo). Standards were purchased from Cayman (Netherlands).

### 2.2. Animal Studies

DBA/1J male mice (6-7 weeks; Charles River Laboratories) were used in this study. Twenty mice were randomly divided in two groups (10 in CIA group, 10 in Ctrl group as healthy control). In the CIA group, immunization with collagen type II will provoke chronic polyarthritis by the induced autoimmune response. Each mouse was intraperitoneally induced (i.p.) with joint cartilage component collagen type II (CII; 100 *μ*g diluted with a 100 *μ*L volume 0.005 M acetic acid) which was extracted from bovine nasal cartilage (Funakoshi Co., Tokyo, Japan) at day 0 (*T* = 0). Thereafter, the CII injection was repeated i.p. on days 14, 28, 42, and 56. In the Ctrl mice, 100 *μ*L of 0.005 M acetic acid alone was administered i.p. on the same days (0, 14, 28, 42, and 56).

Next, to all experimental mice, 5 mg of lipopolysaccharide from* E. coli* 011:B4 (Chondrex, Redmond, USA) dissolved in 100 *μ*L phosphate buffered saline (PBS) was given i.p. immediately after each injection of CII. In the Ctrl group, 100 *μ*L PBS was similarly administered as a control. This protocol for arthritis induction is well established and extensively described [30]. All animals were maintained in a temperature and light controlled environment with free access to standard rodent chow and water. From day 71 to day 75, blood was taken from each animal of both groups (CIA mice (CIA1) died when sampling, leaving 9 animal blood samples in the CIA group) and collected in precooled tubes containing EDTA (ethylenediaminetetraacetic acid) as coagulant (BD Vacutainer, Plymouth, UK). After centrifugation at 3000 g for 10 minutes, the EDTA-plasma was collected and aliquots were stored at −80°C until further processing.

### 2.3. Ethics Statement

This study was carried out in strict accordance with the recommendations in the Guide for the Care and Use of Laboratory Animals of the National Institutes of Health. The experiments were performed with the approval of the Tohoku Institute of Technology Research Ethics Committee, Sendai, Japan (approval date 18 January 2009).

### 2.4. Oxylipin HPLC-MS/MS Analysis on Study Mouse Samples

The details of extraction and analysis of oxylipins species were adapted for the analysis of mouse plasma from a previously described oxylipin profiling method [31]. Antioxidant mixture (5 *μ*L) (0.4 mg/mL BHT and 0.4 mg/mL EDTA mixed with volume ratio 1 : 1) and a mixture of internal-standard mixtures (ISTDs) (5 *μ*L, 1000 nM) were added into each 50 *μ*L aliquot of mouse plasma. Subsequently the samples were loaded on the activated SPE plates (Oasis-HLB 96-well plates, 60 mg, 30 *μ*m) and eluted using ethyl acetate (1.5 mL). The dried eluate was redissolved in 50 *μ*L acetonitrile/methanol (50 : 50 v/v) and 5 *μ*L was analyzed by HPLC (Agilent 1290, San Jose, CA, USA) on an Ascentis Express column (2.1 × 150 mm, particle size of 2.7 *μ*m) coupled to electrospray ionization on a triple quadrupole mass spectrometer (Agilent 6490, San Jose, CA, USA). Performance characteristics for the adapted method including recovery, linearity (*R* ^2^), linear dynamic range, and sensitivity (LOD/LOQ) were evaluated in a separate validation experiment and the results were comparable to those published before for human plasma by Strassburg et al. [31]. The data is included in the Supplementary Material (Table S1, Figure S1, available online at http://dx.doi.org/10.1155/2015/543541).

### 2.5. Data Processing and Statistical Analysis

Peak areas were exported from Mass Hunter software (Agilent Technologies, version B.05.01) and ratios to internal standards were computed (target compounds/ISTDs). Subsequently, an in-house developed QC tool [32, 33] was used to correct for instrument drift and batch effects. The reliability of the measurements was assessed by calculating the reproducibility of each metabolite in a QC pool which was measured after every 10 samples. Oxylipins which met the criteria RSD-QC lower than 35% were included in the final list for the further statistical analysis. Data were log transformed (Glog) and scaled by the standard deviation (autoscaling) in order to get a normal distribution [34, 35]. Univariate analysis (two-tailed unpaired Student's *t*-test) was employed to evaluate significant differences between groups for each metabolite (determined by *p* < 0.05). Principal component analysis (PCA) and partial least square discriminant analysis (PLSDA) were performed to further investigate the discrimination oxylipins between the two groups using tools provided in the metaboanalyst software package (http://www.metaboanalyst.ca/) [36]. Cross-validation was used in order to validate the performance of the PLS-DA model [37]. A permutation test with 100 iterations was performed to estimate the null distribution, by randomly permuting the class labels of the observations. *p* values of each pair of comparison in the permutation test were calculated to evaluate the null hypotheses. To select the potential important metabolites which contribute to group separation, Variable Importance in the Projection (VIP) scores based on PLS-DA analysis were used. The higher the VIP score of a metabolite is, the greater its contribution in the group clustering will be. VIP scores higher than 0.8 are considered as meaningful. Variables with VIP score higher than or equal to 1 were considered as significant important features [38, 39].

## 3. Results 

In this study, the relative concentrations of a panel of oxylipins were determined in control and CIA mice. When evaluating the results from the LC-MS/MS analysis, lower responses of ISTDs peak areas were found in two samples, which lead to an extreme high peak area ratio compared with other study samples. Therefore, these two outliers from Ctrl group were excluded from statistical analysis. The list of detected endogenous oxylipins in mice plasma assigned by their precursors is given in Table 1 (details in supplementary table).

### 3.1. Univariate and Multivariate Analysis Results

**Figure 1:**
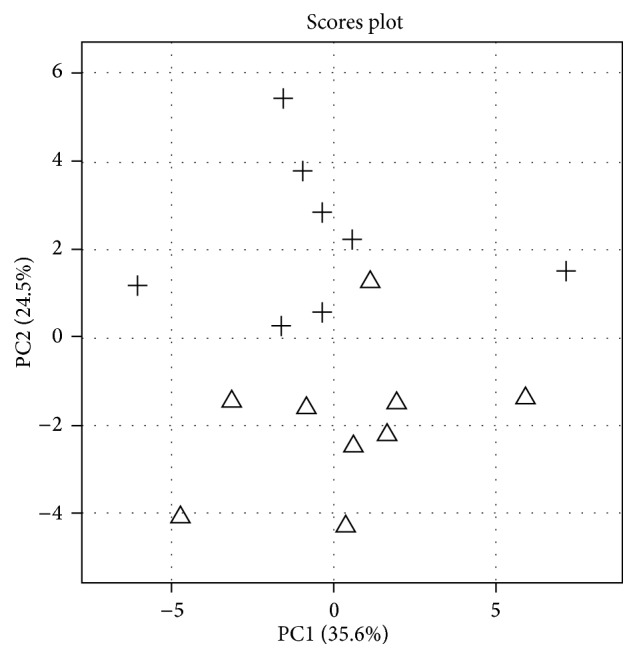
PCA plot of oxylipin data in study mice plasma. PCA score plot of plasma oxylipin data from all study samples revealed general clusters in CIA mice samples and Ctrl samples. The individual samples were marked with “△” or “+” to show the group (CIA versus Ctrl) clustering.

From the QC corrected data, a total of 30 unique oxylipins out of a target list of 110 oxylipins included in the metabolomics platform met the criteria RSD-QC <35%. In order to generally visualize the variance of the samples, a principal components analysis (PCA), as an unsupervised multivariate analysis approach, was performed using these oxylipins.  Figure 1 displays the PCA results in the form of a score plot. The first two principal components accounted for 60.1% of the total variance (PC1 35.6% and PC2 24.5%, resp.), which means the model explains well the variance of the samples. The score plot showed a natural distribution of samples between the CIA group and Ctrl group (consisting of the symbols “△” or “+” plots). All 8 samples (100%) of Ctrl group clustered in PCA. Eight out of 9 mice (88.9%) of CIA group clustered as well, while one sample in CIA group was misclassified and clustered within the Ctrl group. This cluster indicates that there are some differences between the samples, which were mainly a reflection of the CIA/Ctrl groups.

**Figure 2:**
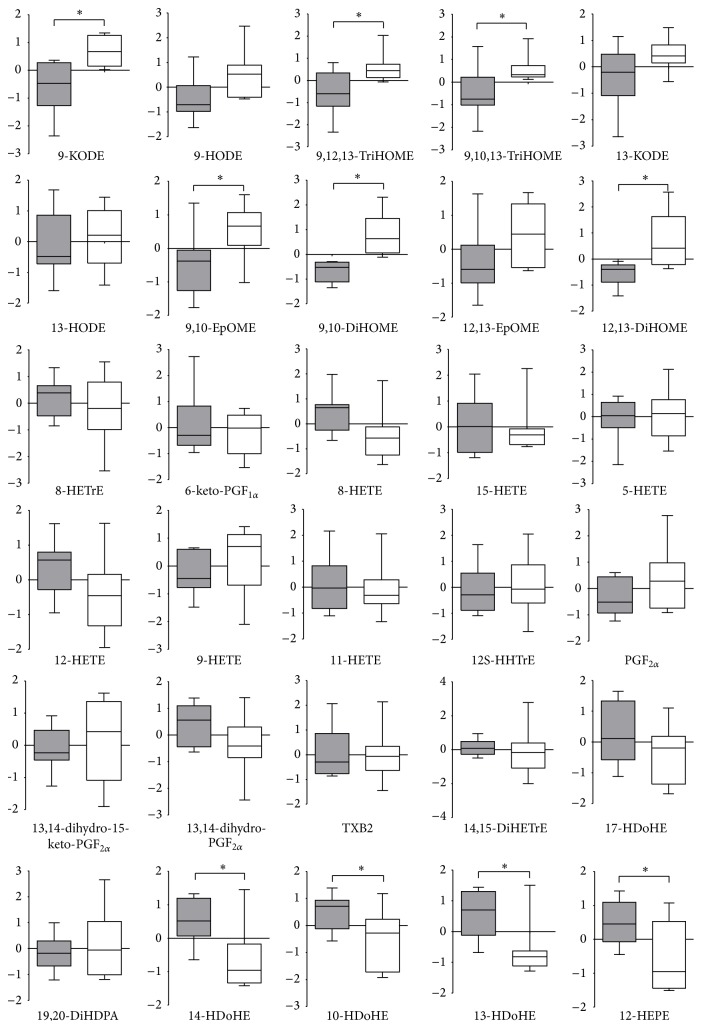
Changes in metabolite levels between Ctrl and CIA mice. Individual metabolite levels for the two groups are illustrated using box-plots with the whisker drawn, after logarithmic transformation for normalization. Boxplot colored: white box: metabolites in Ctrl group; grey box: metabolites in CIA group. The metabolites which differed significantly based on Student's *t*-test (*p* < 0.05) are marked with “*∗*”.

Determining the oxylipin species responsible for the differences between the CIA and Ctrl group is key to unraveling the biological role of this class of compounds in RA. Student's *t*-test is one of the most widely used methods to determine the statistical significance. In order to understand which of the detected oxylipins showed significant differences between the two groups, an unpaired Student's *t*-test analysis was evaluated in each individual metabolite. From the *t*-test, 10 out of the 30 detected oxylipins (percentage of 33.3%) showed significant differences (*p* < 0.05), namely, 9,10-DiHOME, 9-KODE, 12,13-DiHOME, 14-HDoHE, 13-HDoHE, 12S-HEPE, 9,12,13-TriHOME, 9,10,13-TriHOME, 9,10-EpOME, and 10-HDoHE. In order to show the effect size and variance among the samples, a comparison of individual metabolite levels measured for CIA and control mice is displayed in Figure 2, in the form of boxplots, with “*∗*” indicating statistical significance between groups. In the boxplot, lines extended from the boxes (whiskers) showed the variabilities outside from the upper and lower quartiles of the data.

Given that compounds which showed nonsignificant changes from univariate approaches (such as *t*-tests) may also contribute to group clustering and provide useful information on biological interpretation, a PLS-DA model as a supervised clustering method was further applied to get a more focused view on the metabolites which contribute to group clustering. A PLS-DA scores plot using two components with total score of 43.5% (component 1 = 24.5%, component 2 = 19%) gives a reasonable group separation (figure in supplementary data). However, this model needs to be validated in order to prevent overfitting. Therefore, cross-validation and permutation test was performed. The predictive accuracy (0.88) accompanied with a goodness of fit *R* ^2^ (0.84) in cross-validation revealed a sound basis for the PLS-DA model. The permutation tests with an average of 4 misclassifications in 100 iterations (*p* = 0.04) showed robustness of the model. Thus classification of groups based on this approach can be considered as significant based on both cross-validation and 100 permutation tests.

For this model, the Variable Importance in the Projection (VIP) score was used to summarize the relative contributions of each individual metabolite to the group separation in the PLS-DA. The VIP score shows 14 variables which contributed to the group clustering (VIP > 1), including 5 upregulated oxylipins (14-HDoHE, 13-HDoHE, 12S-HEPE, 10-HDoHE, and 8-HETE) and 9 downregulated oxylipins (9,10-DiHOME, 9-KODE, 12,13-DiHOME, 9,12,13-TriHOME, 9,10,13-TriHOME, 9,10-EpOME, 9-HODE, 13-KODE, and 12,13-EpOME). The top ten of them are also detected in univariate *t*-test results, which confirmed the importance of these oxylipins.

Given that the oxylipins 13,14-dihydro-PGF_2*α*_ and 12-HETE have been implicated in inflammatory regulation in disease and also given that they showed a meaningful VIP score close to 1 (0.96, 0.95, resp.) with increasing trend in the CIA group, changes in these metabolites can provide insight in the biological interpretation for CIA and are included in further biological interpretation. The detailed pieces of information of *p* value from Student's *t*-test, VIP scores from PLS-DA, and their direction of regulation are shown in Table 1.

### 3.2. Physiological Pathways of Altered Oxylipins

**Figure 3:**
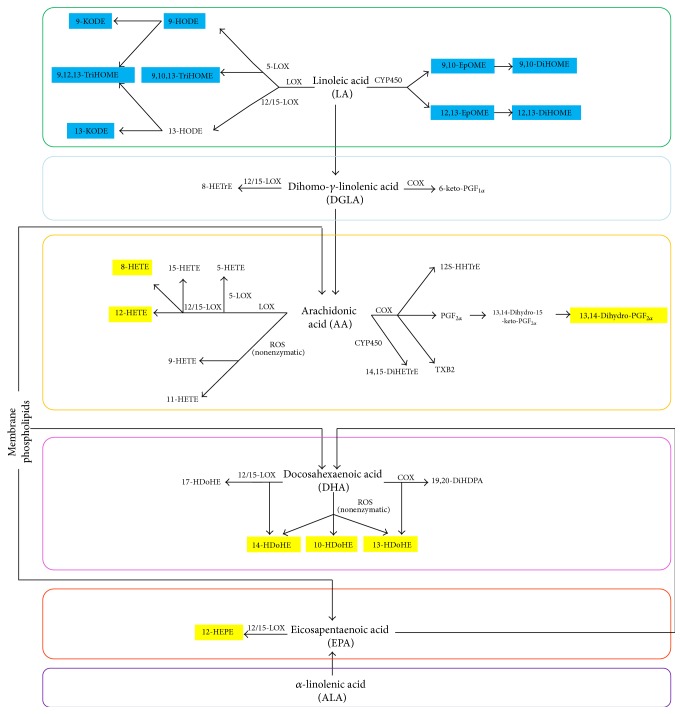
Overview of regulations of oxylipins in CIA mice compared with Ctrl, including metabolic pathways. Metabolites detected in mice plasma are grouped by metabolic pathways. Important metabolites which contribute most to group clustering based on PLS-DA are colored: yellow box: upregulated in the CIA group; blue box: downregulated in the CIA group.

We grouped the detected oxylipins by their metabolic pathways in order to illustrate their biological roles in Figure 3. Color is used to indicate the up/downregulation (marked in yellow/blue boxes) in the CIA group.

Among these colored 16 metabolites, all the 9 downregulated oxylipins (9,10-DiHOME, 9-KODE, 12,13-DiHOME, 9,12,13-TriHOME, 9,10,13-TriHOME, 9,10-EpOME, 9-HODE, 13-KODE, and 12,13-EpOME) are derived via the LA group; 3 upregulated oxylipins (8-HETE, 13,14-dihydro-PGF_2*α*_, and 12-HETE) are derived from AA; 3 upregulated oxylipins (14-HDoHE, 13-HDoHE, and 10-HDoHE) are derived from DHA; and 1 upregulated oxylipin (12S-HEPE) is produced from EPA.

## 4. Discussion

**Figure 4:**
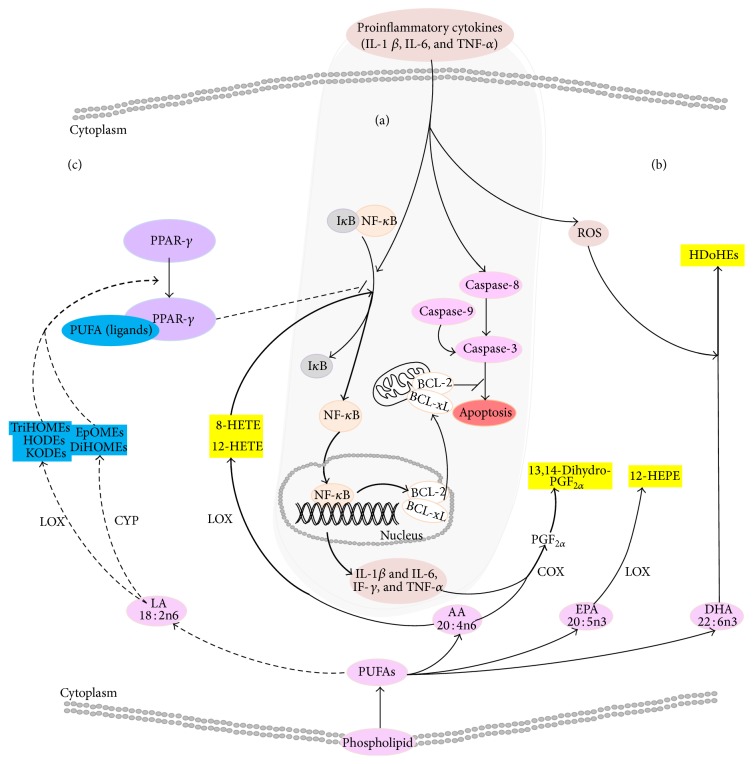
A systematic autoimmune activation in RA. Appearance of proinflammatory cytokines (IL-1*β* and IL-6, TNF*α*) as well as the appearance of ROS in RA. The cytokines normally induce the apoptosis via the caspase pathway but also inhibit apoptosis through degradation I*κ*B activating nuclear factor-*κ*B (NF-*κ*B), which consequently translocate to the nucleus upregulating the antiapoptotic genes (BcL2 and BcL-xL). The activated NF-*κ*B then can also further enhance the production of proinflammatory cytokines and chemokines as well as COX-II enzyme. (b) Upregulated oxylipin response. During RA increased levels of AA derived prostaglandins and HETEs are detected. 8- and 12-HETE are able to activate NF-*κ*B exasperating RA. Due to increased levels of ROS, DHA derived peroxidation products are also found. (c) Dysregulated anti-inflammatory response. LA derived oxylipins including HODEs, KODEs, TriHOMEs, DiHOMEs, and EpOMEs are ligands of peroxisome proliferator-activated receptor- (PPAR-) *γ*. Due to decreased levels of these anti-inflammatory oxylipins, the ability of PPAR-*γ* to inhibit the activation of NF-*κ*B and indirectly affect apoptosis is diminished.

Inflammation is a self-limiting innate mechanism under complex regulation with the purpose to recruit leukocytes and plasma proteins, trafficking these to the site of infection or tissue damage, supporting a robust adaptive immune response and subsequent resolution [40]. RA is the consequence of a systemic autoimmune activation/response within the synovial fluid in the joint triggering a dysregulated chronic inflammatory response, of which the exact underlying pathogenic mechanisms still remain largely unclear. RA is characterized with a strong inflamed cytokine phenotype with elevated levels of IL-1*β*, IL-6, and TNF*α* as well as increased levels of ROS [18, 41, 42], seen in Figure 4(a). Perturbations related to TNF*α* activation of the NF-*κ*B pathway inhibiting apoptosis in activated antigen-presenting cells including neutrophils, macrophages, fibroblast-like cells, and B-cells form the general accepted pathological basis of RA [9, 10, 43, 44]. Hence we applied a comprehensive oxylipin metabolomics platform to the plasma of DBA/1J mice induced by a coadministration of type II collagen with lipopolysaccharide, to elucidate the role of these potent inflammatory mediators in RA.

We detected an increased proinflammatory oxylipin response, which can be attributed to the activation of NF-*κ*B and increased ROS (Figure 4(b)). NF-*κ*B is the transcription factor for COX-II, and its activation during RA [45, 46] can explain the increased levels of the COX derived prostaglandin F_2*α*_ measured via its downstream product 13,14-dihydro-PGF_2*α*_ in CIA mice [47, 48]. Several hydroxyl-fatty acids were also implicated as role players in the chronic inflammatory phenotype of RA. Due to two possible de novo synthesis routes for hydroxyl-fatty acids, it implicates increased LOX activity concurrently with elevated oxidative stress within CIA mice [24–27]. Increased 12-LOX signaling mediators included 8-HETE and 12-HETE supporting a proinflammatory milieu [49, 50]. In an oral tolerance test in CIA rats, Ding et al. [51] measured elevated levels of EPA-derived 18-HEPE, while we detected increased level of a similar metabolite 12-HEPE. Overexpression of 12-LOX in RA has been published by Liagre and Kronke [52, 53], which can further mediate the activation of NF-*κ*B [54–56], indicating the chronic nature of RA. However, 8-HETE, 12-HETE, and 12-HEPE together with the docosahexaenoic acid derived HDOHEs also provide a readout for ROS induced biologically active lipid peroxidation products [24–27]. Oxidative stress leading to increased free radicals as well as ROS levels has been reported in RA by Ozkan et al. [18], supporting this finding.

Alongside the increased pro-inflammatory oxylipins, we also identified significantly decreased LA derived oxylipins in CIA mice plasma. The decreased LA cytochrome P450 products (EpOMEs, DiHOMEs) and LA LOX products (TriHOMEs) implicate a fatty acid precursor perturbation and/or a possible oxylipin enzymatic impairment in RA. AA is the ELOVL mediated elongation product of LA, and the detected increasing trend in AA derived oxylipins indicate sufficient CYP and LOX activity to rule out enzyme activity as the cause of the LA oxylipin reductions. In addition, these LA derived oxylipins as well as the decreased HODEs and KODEs are ligands for nuclear hormone receptor peroxisome proliferator-activated receptor-gamma (PPAR-*γ*) activation [57–63], shown in Figure 4(c). PPAR-*γ* are anti-inflammatory regulators of immune cells and can inhibit the activation of NF-*κ*B [44, 46, 61, 62, 64–70]. Therefore, the decreased LA-derived oxylipins and PPAR-*γ* ligands indicate a perturbation in mechanisms related to the resolution of inflammation, unable to inhibit NF-*κ*B activation and its downstream inhibition of apoptosis.

As discussed above, our detected oxylipins indicate insufficient PPAR-*γ* ligands, as well as mechanisms leading to the activation of NF-*κ*B, supporting and enhancing our understanding of the inhibition of apoptosis in CIA mice. Apoptosis plays an important role leading to the phagocytic clearances of damage cells stifling the development of chronic inflammation and autoimmunity [71]. The inhibition of apoptosis prevents the silencing of activated leukocytes, dysregulating clearance mechanisms contributing to chronic autoimmune inflammation in RA [72].

## 5. Conclusion

Using our comprehensive oxylipin method we were able to show that the CIA mice had an arachidonic acid dependent increased proinflammatory profile, with increased levels of oxidative stress. Several studies have been published advocating anti-inflammatory diets (the restriction of AA in the diet), leading to therapeutic benefits and ameliorating RA [73]. We also detected a significant decrease in potent anti-inflammatory oxylipins derived from linoleic acid capable of signaling via PPAR-*γ* to inhibit the activation of NF-*κ*B, namely, the molecular basis for RA. Interestingly, PPAR-*γ* has been identified and reported as a therapeutic agent for arthritis [74]. The reduced levels of linoleic acid derived oxylipins implicated fatty acid precursor pools, shedding light on the unexplored routes of fatty acid elongation pathways in the pathogenicity of RA, and need further work. As additional metabolites have been reported to play a role in RA, a systems biology approach would complement the study of systematic autoimmune induced rheumatoid arthritis.

## Supplementary Material

The supplementary material provides the methodology of oxylipin extraction and detection and reports performance characteristics of this method. Detailed results from supervised. PLS-DA analysis and VIP scores are also provided in order to demonstrate the important contributions of significant oxylipins to the group clusters.

## Figures and Tables

**Table 1:** List of oxylipins detected in mice plasma, measured by multiple reaction monitoring (precursor ions → product ions) in LC-MS/MS analysis.

Compounds	MS transitions (*m*/*z*)	*p* value	VIP	Regulation	Pathway
*LA*					
9,10-DiHOME	313.2 → 201.1	0.0002	1.86	↓	CYP450
12,13-DiHOME	313.2 → 183.2	0.006	1.51	↓	CYP450
9,10-EpOME	295.2 → 171.2	0.028	1.27	↓	CYP450
12,13-EpOME	295.2 → 195.2	0.096	1.00	↓	CYP450
9-KODE	293.2 → 185.2	0.003	1.61	↓	5-LOX
9,12,13-TriHOME	329.2 → 211.2	0.017	1.36	↓	5-LOX
9,10,13-TriHOME	329.2 → 171.1	0.026	1.29	↓	5-LOX
9-HODE	295.2 → 171.1	0.052	1.14	↓	5-LOX
13-KODE	293.2 → 113.1	0.082	1.04	↓	12/15-LOX
13-HODE	295.2 → 195.2	0.733	0.21	—	12/15-LOX
*EPA*					
12-HEPE	317.2 → 179.1	0.016	1.37	↑	12/15-LOX
*DHA*					
14-HdoHE	343.2 → 205.0	0.010	1.45	↑	ROS
13-HdoHE	343.2 → 281.0	0.012	1.42	↑	ROS
10-HdoHE	343.2 → 153.0	0.035	1.23	↑	ROS
17-HdoHE	343.2 → 281.3	0.173	0.83	—	12/15 LOX
19,20-DiHDPA	361.2 → 273.3	0.509	0.41	—	CYP450
*DGLA*					
6-keto-PGF_1*α*_	369.2→ 163.1	0.390	0.53	—	COX
8-HETrE	321.3 → 303.0	0.469	0.45	—	12/15 LOX
*AA*					
8-HETE	319.2 → 155.1	0.074	1.06	↑	12/15-LOX
12-HETE	319.2 → 179.2	0.116	0.95	↑	12/15 LOX
15-HETE	319.2 → 219.2	0.770	0.18	—	12/15-LOX
5-HETE	319.2 → 115.1	0.713	0.23	—	5-LOX
13,14-dihydro-PGF_2*α*_	355.2 → 275.3	0.112	0.96	↑	COX
PGF2_*α*_	353.2 → 193.1	0.176	0.82	—	COX
13,14-dihydro-15-keto-PGF_2*α*_	353.2→ 183.1	0.618	0.31	—	COX
12S-HHTrE	279.2 → 179.2	0.733	0.21	—	COX
TXB2	369.2 → 169.1	0.900	0.08	—	COX
14,15-DiHETrE	337.2 → 207.2	0.662	0.27	—	CYP450
9-HETE	319.2 → 167.1	0.408	0.51	—	ROS
11-HETE	319.2 → 167.1	0.820	0.14247	—	ROS

The oxylipins are grouped based on the original polyunsaturated fatty acid precursor: linoleic acid (LA), eicosapentaenoic acid (EPA), docosahexaenoic acid (DHA), dihomo-*γ*-linolenic acid (DGLA), and arachidonic acid (AA). Their metabolic pathways include enzymatic pathways: cyclooxygenase (COX), lipoxygenase (LOX), cytochrome P450 (P450), and nonenzymatic reactive oxygen species (ROS) pathway. The significance of changes between two groups was illustrated by *p* value from univariate test (Student's *t*-test) and VIP score from multivariate test (PLS-DA). The important regulations in the CIA group were marked with “↓” or “↑” selected based on VIP scores.

↓: downregulated in CIA group.

↑: upregulated in CIA group.

## Abbreviations

AA: Arachidonic acid
ALA: *α*-arachidonic acid
CIA: Collagen induced arthritis
CII: Collagen type II
COX: Cyclooxygenase
CYP 450: Cytochrome P450 epoxygenases
DGLA: Dihomo-*γ*-linolenic acid
DHA: Docosahexaenoic acid
DiHETrE: Dihydroxyeicosatrienoic acid
DiHOME: Dihydroxyoctadeca (mono) enoic acid
EPA: Eicosapentaenoic acid
EpETrE: Epoxyeicosatrienoic acids
EpOME: Epoxyoctadecenoic acid
HDoHE: Hydroxydocosahexaenoic acid
HEPE: Hydroxyeicosapentaenoic acid
HETE: Hydroxyeicosatetraenoic acid
HETrE: Hydroxyeicosatrienoic acid
HHTrE: Hydroxyheptadecatrienoic acid
HODE: Hydroxyoctadecadienoic acid
HOTrE: Hydroxyoctadecatrienoic acid
ISTDs: Internal standards
KETE: Ketoeicosatetraenoic acid
KODE: Ketooctadecadienoic acid
LA: Linoleic acid
LOX: Lipoxygenase
LPS: Lipopolysaccharide
LT: Leukotrienes
LX: Lipoxins
NF-*κ*B: Nuclear factor-kappa B
PG: Prostaglandin
PPAR: Peroxisome proliferator-activated receptor
RA: Rheumatoid arthritis
ROS: Reactive oxygen species
sEH: Soluble epoxide hydrolase
TNF: Tumor necrosis factor
TriHOME: Trihydroxyoctadecenoic acid
TX: Thromboxane.
