# Supplementary material for: Collagen Induced Arthritis in DBA/1J Mice Associates with Oxylipin Changes in Plasma
Source: Mediators Inflamm. 2015 Oct 29;2015:543541. doi: 10.1155/2015/543541 (PMC4641941; doi:10.1155/2015/543541)
Supplement: Supplementary file 1 — The supplementary material provides the methodology of oxylipin extraction and detection and reports performance characteristics of this method. Detailed results from supervised. PLS-DA analysis and VIP scores are also provided in order to demonstrate the important contributions of significant oxylipins to the group clusters. [file 543541.f1.pdf]

Supplementary figures

Figure S1. Recovery(%)of 15 deuterated oxylipins ISTDs . Oxylipins recovery for SPE extraction method was displayed in forms of percentage value.

Figure S2. Score plot of PLS-DA based on metabolic profiling in CIA model group and Ctrl group.

Figure S3 Variable Importance in the Projection(VIP) scores of detected oxylipins based on PLS-DA.

Fourteen oxylipins showed VIP score higher than 1, while another to are extremely closer to 1(0.96 and 0.95 respectively). The regulation information of increase (■) and decrease (□) are given in the right side of the figure.

Figure S1. Recovery(%)of 15 deuterated oxylipins ISTDs.

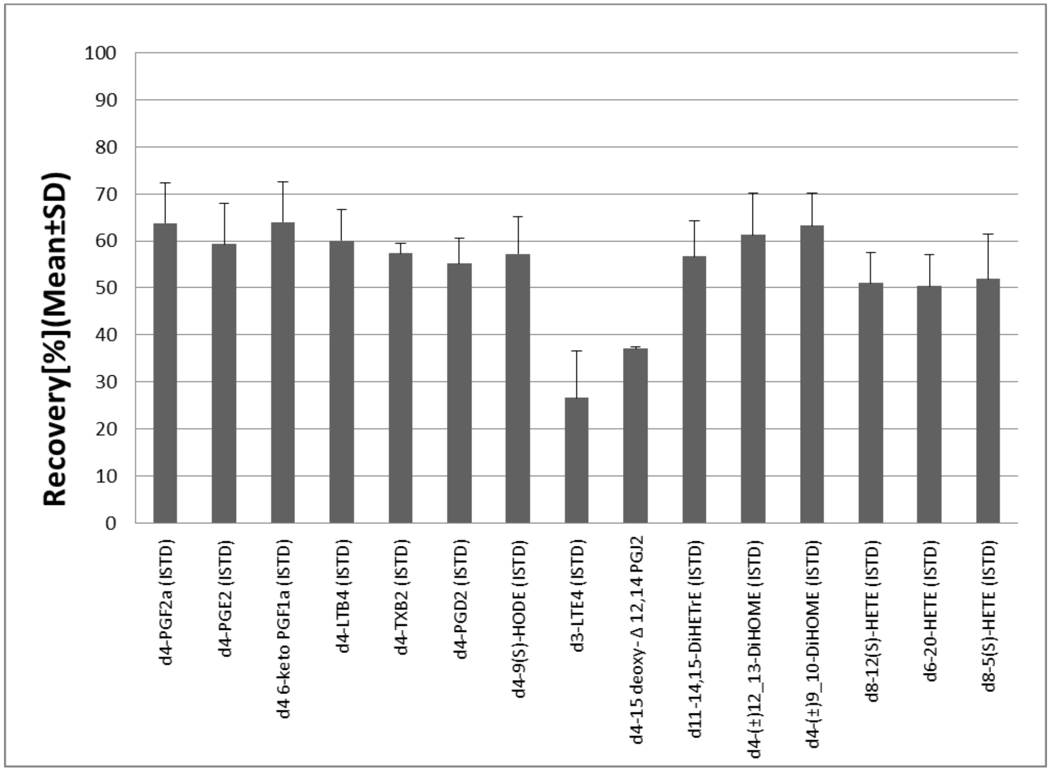

Figure S2. Score plot of PLS-DA based on metabolic profiling in CIA model group and Ctrl group.

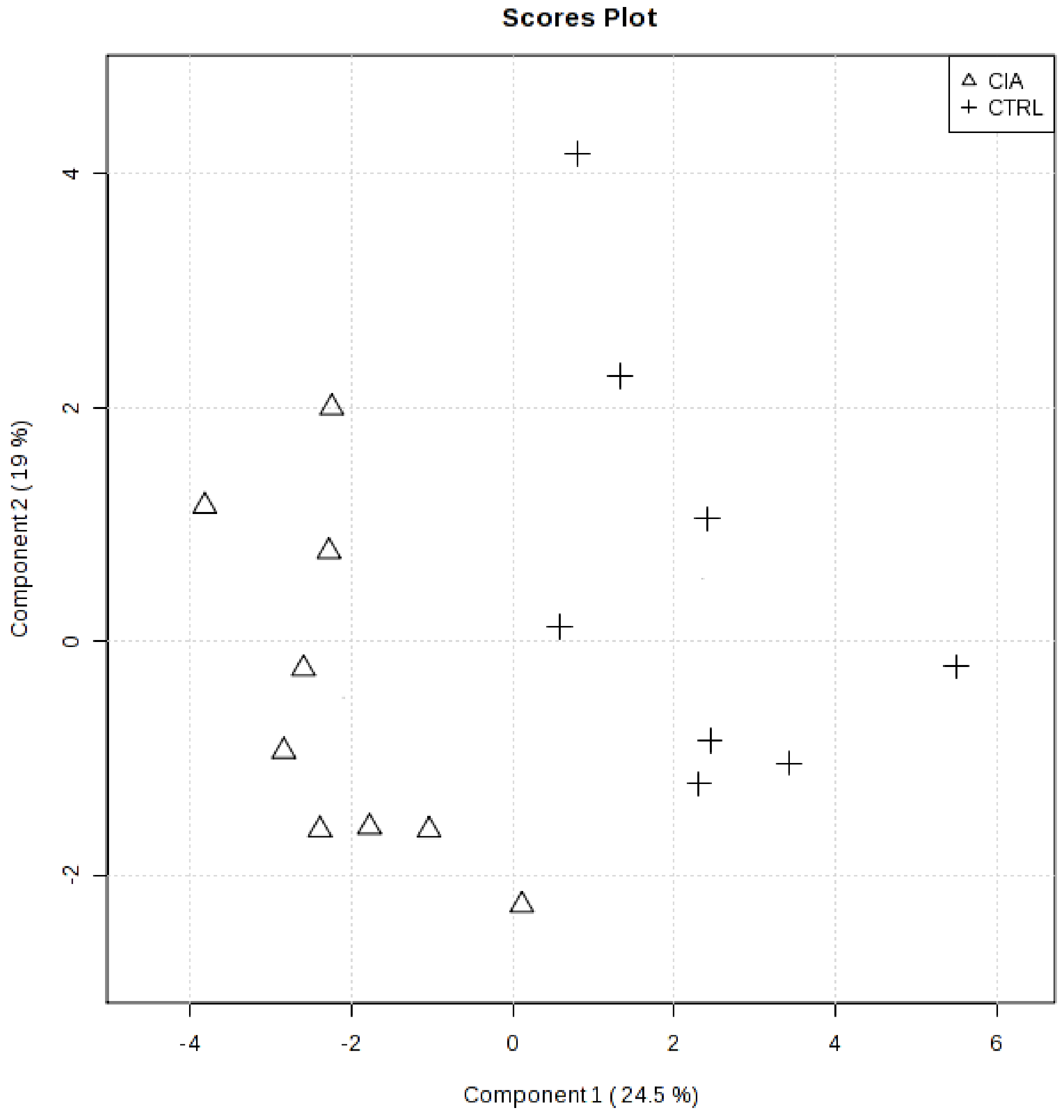

Figure S3. Variable Importance in the Projection(VIP) scores of detected oxylipins based on PLS-DA.

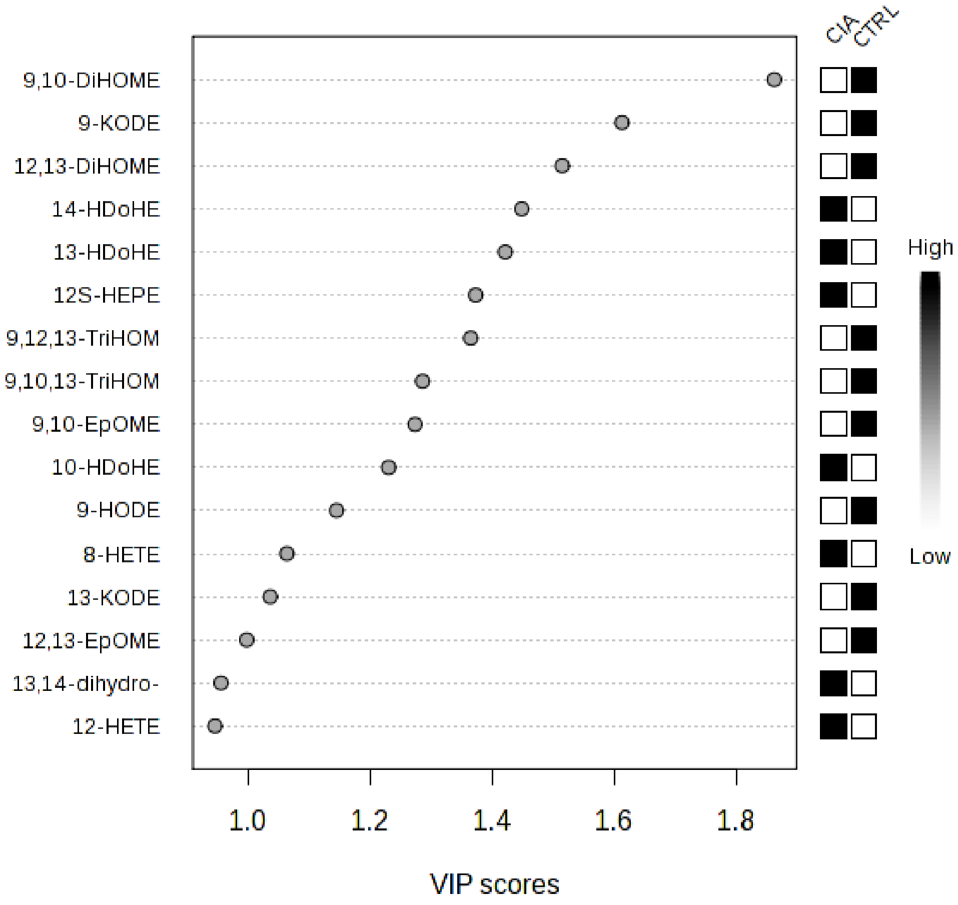

Supplementary tables

Table S1. Linearity(R^2), reproducibility(RSD), Limitation of detection(LOD) and quantitation(LOQ) of LC/ESI-MS/MS for oxylipins detected in mice plasma.

^: oxylipins were also detected in the study samples.

Table S2. Details of detected oxylipins in mice plasma using LC-MS/MS analysis. The table contains 30 oxylipins detected in study mice plasma, with their information of Mass (precursor ions accompanied with product ions) as well as the selected internal standard for ratio calculation and correction, ranked by their retention time. For oxylipins platform, RSD is acceptable if less than 35% because of the less stability and low concentration levels of oxylipins. 30 oxylipins were reliable with RSD <35% .

Table S1. Linearity(R^2), reproducibility(RSD), Limitation of detection(LOD) and quantitation(LOQ) of LC/ESI-MS/MS for oxylipins detected in mice plasma.

Oxylipins	Chemical class	R^2	RSD[%]	LOD[nM]	LOQ[nM]
**AA**					
12S-HHTrE ^	Alcohols	0.999	12	1	3.5
20-HETE	Alcohols	0.999	11	6.1	20.2
15-HETE^	Alcohols	0.904	26	2.6	8.8
11-HETE^	Alcohols	0.963	12	0.6	1.8
12-HETE ^	Alcohols	0.794	7	15.6	52.1
8-HETE^	Alcohols	0.95	30	13.1	43.5
9-HETE ^	Alcohols	0.932	19	33.9	113
5-HETE^	Alcohols	0.82	17	5.2	17.4
5S,6R-LipoxinA4	Diols	0.994	4	1.5	5.1
5S,6S-Lipoxin A4	Diols	0.996	5	4.4	14.5
6-trans-LTB4	Diols	0.99	7	8.3	27.5
LTB4	Diols	0.999	12	0.4	1.3
14,15-DiHETrE ^	Diols	1	9	0.4	1.4
11,12-DiHETrE	Diols	0.999	14	1.6	5.5
8,9-DiHETrE	Diols	0.999	17	0.7	2.4
5,6-DiHETrE	Diols	0.999	5	18.8	62.6
14,15-EpETrE	Epoxides	0.847	7	3.7	12.4
5,6-EpETrE	Epoxides	0.999	8	3.9	12.8
12S-HpETE	Hydroperoxides	0.902	10	116.7	389.1
5S-HpETE	Hydroperoxides	1	27	0.4	1.3
15-KETE	Ketones	0.998	32	4.8	16
5-KETE	Ketones	0.985	32	39.1	130.4
8-iso-PGF2a	Prostanoids/throboids	1	13	0.5	1.7
5-iPF2a-VI	Prostanoids/throboids	1	10	0.1	0.3
TXB2 ^	Prostanoids/throboids	0.999	10	1.3	4.5
PGF2a ^	Prostanoids/throboids	0.999	11	0.9	2.8
PGE2	Prostanoids/throboids	0.998	12	1.9	6.2
11beta-PGE2	Prostanoids/throboids	0.998	6	2.5	8.5
13,14-dihydro-PGF2a ^	Prostanoids/throboids	1	13	4.1	13.8
13,14-dihydro-15-keto-PGF2a^	Prostanoids/throboids	1	9	1.94	6.48
PGA2	Prostanoids/throboids	0.998	8	2.3	7.7
PGJ2	Prostanoids/throboids	1	6	0.03	0.1
d12-PGJ2	Prostanoids/throboids	0.995	10	2.3	7.6
PGD2	Prostanoids/throboids	0.996	6	2.5	8.5
HepoxilinA3	Prostanoids/throboids	0.992	7	57.7	192.3
**ALA**					
9-HOTrE	Alcohols	0.998	12	0.3	1
12,13-DiHODE	Diols	0.993	13	54.5	181.8
**DGLA**					
15S-HETrE	Alcohols	0.998	27	1.3	4.3
8-HETrE^	Alcohols	0.979	15	2.8	9.2
5-HETrE	Alcohols	0.999	23	1.6	5.4
6-keto-PGF1a^	Prostanoids/throboids	1	15	1.08	3.62
**DHA**					
17-HDoHE^	Alcohols	0.988	10	0.4	1.2
20-HDoHE	Alcohols	0.993	14	4.8	15.8
16-HDoHE	Alcohols	0.983	13	7.6	25.5
13-HDoHE^	Alcohols	0.977	11	113.9	379.6
14-HDoHE^	Alcohols	0.900	9	13.7	45.7
10-HDoHE ^	Alcohols	0.974	13	8.8	29.3
7-HDoHE	Alcohols	0.991	9	0.1	0.4
11-HDoHE	Alcohols	0.986	9	3.6	11.8
4-HDoHE	Alcohols	0.976	22	29.6	98.5
8-HDoHE	Alcohols	0.92	19	14	46.8
10S,17S-DiHDoHE	Diols	1	4	1.6	5.4
19,20-DiHDPA ^	Diols	1	19	3.6	11.9
19,20-EpDPE	Epoxides	0.984	16	95.6	318.7
**EPA**					
18-HEPE	Alcohols	0.991	19	29.9	99.7
15-HEPE	Alcohols	0.999	25	7.6	25.3
12-HEPE ^	Alcohols	0.939	11	12.3	40.8
9-HEPE	Alcohols	0.995	15	4.8	16.1
5-HEPE	Alcohols	0.999	25	1.2	4
8S,15S-DiHETE	Diols	0.832	3	40.3	134.4
5S,15S-DiHETE	Diols	0.998	4	8.2	27.2
5S,6S-DiHETE	Diols	1	9	3.0	10.1
**LA**					
13-HODE ^	Alcohols	0.884	9	2.7	9.2
9-HODE ^	Alcohols	0.903	8	1.6	5.3
12,13-DiHOME ^	Diols	1	3	0.9	3
9,10-DiHOME ^	Diols	0.999	5	0.6	2.1
12,13-EpOME ^	Epoxides	1	9	1.6	5.3
9,10-EpOME ^	Epoxides	0.999	26	1.1	3.7
13-KODE^	Ketones	0.996	6	1.4	4.7
9-KODE^	Ketones	0.998	9	0.6	1.9
9,12,13-TriHOME ^	Triols	0.994	8	1	3.3
9,10,13-TriHOME ^	Triols	0.996	9	2.7	9.1

Table S2. Details of detected oxylipins in study mice plasma using LC-MS/MS analysis.

Compounds	Lipid Maps ID	Mass (precursor ion → product ions)	Retention Time	Internal standard	Precision RSD[%]	FA group	Chemcal class
6-keto-PGF1a	LMFA03010001	369.2-> 163.1	5.21	d4 6-keto PGF1a	6.9	DGLA	Prostanoids
TXB2	LMFA03030002	369.2 -> 169.1	7.05	d4-TXB2	0.4	AA	throboids
9,12,13-TriHOME	LMFA02000014	329.2 -> 211.2	7.10	d4-9(S)-HODE	26.1	LA	Tiols
PGF2a	LMFA03010002	353.2 -> 193.1	7.20	d4-PGF2a	7.7	AA	Prostanoids
9,10,13-TriHOME	LMFA02000168	329.2 -> 171.1	7.24	d4-9(S)-HODE	19.7	LA	Tiols
13,14-dihydro-PGF2a	LMFA03010079	355.2 -> 275.3	8.08	d4-PGF2a	8.7	AA	Prostanoids
13,14-dihydro-15-keto-PGF2a	LMFA03010027	353.2-> 183.1	8.67	d4-PGF2a	5.6	AA	Prostanoids
12,13-DiHOME	LMFA01050351	313.2 -> 183.2	12.07	d4-(±)12_13-DiHOME	14.0	LA	Diols
9,10-DiHOME	LMFA01050350	313.2 -> 201.1	12.46	d4-(±)9_10-DiHOME	34.5	LA	Diols
19,20-DiHDPA	LMFA04000043	361.2 -> 273.3	12.87	d11-14,15-DiHETrE	23.3	DHA	Diols
14,15-DiHETrE	LMFA03050010	337.2 -> 207.2	12.91	d11-14,15-DiHETrE	27.6	AA	Diols
12S-HHTrE	LMFA03050002	279.2 -> 179.2	12.99	d8-12(S)-HETE	30.4	AA	Alcohols
12-HEPE	LMFA03070008	317.2 -> 179.1	14.91	d8-12(S)-HETE	9.4	EPA	Alcohols
13-HODE	LMFA01050349	295.2 -> 195.2	15.37	d4-9(S)-HODE	5.6	LA	Alcohols
9-HODE	LMFA01050278	295.2 -> 171.1	15.50	d4-9(S)-HODE	6.5	LA	Alcohols
15-HETE	LMFA03060001	319.2 -> 219.2	15.83	d8-5(S)-HETE	21.6	AA	Alcohols
13-KODE	LMFA02000016	293.2 -> 113.1	15.96	d4-9(S)-HODE	4.3	LA	Ketones
17-HDoHE	LMFA04000032	343.2 -> 281.3	16.10	d8-12(S)-HETE	10.7	DHA	Alcohols
11-HETE	LMFA03060028	319.2 -> 167.1	16.23	d8-12(S)-HETE	10.1	AA	Alcohols
14-HDoHE	LMFA04000030	343.2 -> 205.0	16.28	d8-12(S)-HETE	4.9	DHA	Alcohols
13-HDoHE	LMFA04000029	343.2 -> 281.0	16.29	d8-12(S)-HETE	7.9	DHA	Alcohols
10-HDoHE	LMFA04000027	343.2 -> 153.0	16.32	d8-12(S)-HETE	26.2	DHA	Alcohols
9-KODE	LMFA01060177	293.2 -> 185.2	16.37	d4-9(S)-HODE	18.3	LA	Ketones
12-HETE	LMFA03060088	319.2 -> 179.2	16.52	d8-12(S)-HETE	13.0	AA	Alcohols
8-HETE	LMFA03060006	319.2 -> 155.1	16.54	d8-5(S)-HETE	6.4	AA	Alcohols
9-HETE	LMFA03060089	319.2 -> 167.1	16.77	d8-12(S)-HETE	12.3	AA	Alcohols
5-HETE	LMFA03060002	319.2 -> 115.1	17.00	d8-5(S)-HETE	18.2	AA	Alcohols
8-HETrE	LMFA03050011	321.3 -> 303.0	17.06	d8-12(S)-HETE	19.6	DGLA	Alcohols
12,13-EpOME	LMFA02000038	295.2 -> 195.2	17.40	d4-(±)12_13-DiHOME	24.3	LA	Epoxides
9,10-EpOME	LMFA02000037	295.2 -> 171.2	17.59	d4-(±)9_10-DiHOME	5.6	LA	Epoxides
